# Content validity of the Familial Chylomicronemia Syndrome Symptoms and Impacts Scale (FCS-SIS) for severe hypertriglyceridemia (sHTG)

**DOI:** 10.1080/1179271X.2026.2672471

**Published:** 2026-05-27

**Authors:** Asia Sikora Kessler, Tammy Michelle Brown, Bonita Basnyat, Aaron Yarlas, Jennifer Dine

**Affiliations:** a Health Economics & Outcomes Research, Ionis Pharmaceuticals Inc., Carlsbad, CA, US; b Patient-Centered Outcomes Assessment, RTI Health Solutions, Durham, NC, US

**Keywords:** Severe hypertriglyceridemia, sHTG, familial chylomicronemia syndrome, FCS, FCS-SIS, content validity

## Abstract

**Objective:**

Severe hypertriglyceridemia (sHTG) and familial chylomicronemia syndrome (FCS) are metabolic conditions associated with an increased risk of adverse health outcomes. The FCS Symptoms and Impacts Scale (FCS-SIS) was developed to assess the symptoms and impacts of FCS. This study assessed the content validity of the 4 FCS-SIS symptom items in adults with sHTG.

**Methods:**

Two iterative rounds of qualitative interviews were conducted with adults (aged ≥ 18 years) who had clinician-confirmed sHTG (triglycerides ≥ 500 mg/dL) and recently experienced ≥ 2 symptoms of sHTG. Participants described their initial experiences and symptoms of sHTG during concept elicitation. Cognitive debriefing of the 4 FCS-SIS symptom items assessed relevance, interpretability, answerability, and meaningful change.

**Results:**

Among 20 participants, the most frequently reported, most bothersome, and most important-to-treat sHTG symptoms were difficulty thinking (n = 20), physical fatigue (n = 20), diarrhea (n = 19), and abdominal pain (n = 18), which the 4 FCS-SIS symptom items assess. Participants reported these items to be easy to understand (n = 20) and, for most items, reported that a 1- to 2-point improvement would be meaningful.

**Conclusion:**

FCS-SIS symptom items are content-valid items for the assessment of sHTG symptoms in adults with sHTG.

## PLAIN LANGUAGE SUMMARY

Severe hypertriglyceridemia (sHTG) and familial chylomicronemia syndrome (FCS) are conditions in which fats are not sufficiently broken down. Patients with these conditions experience similar symptoms. A tool called the FCS-Symptom and Impact Scale (FCS-SIS) can be used to measure the symptoms of these conditions. This tool was originally developed for patients with FCS, but it is now used in clinical trials of sHTG. The 4 symptoms measured by the FCS-SIS are stomach pain, fatigue, difficulty thinking, and diarrhea. Even though patients with sHTG can experience any of these 4 symptoms, the appropriateness of the FCS-SIS for measuring sHTG symptoms has not been studied. In this study, we conducted interviews with adults with sHTG to assess whether the 4 symptoms measured by the FCS-SIS are relevant to them. We found that these 4 symptoms were the most frequently reported, the most bothersome, and the most important-to-treat symptoms by these adults with sHTG. Most of the adults understood what these symptoms are and found them to be relevant to their sHTG symptom experience. Therefore, we found the FCS-SIS to be a relevant tool for measuring sHTG symptoms.

## Introduction

Severe hypertriglyceridemia (sHTG) and familial chylomicronemia syndrome (FCS) are rare metabolic conditions characterized by hypertriglyceridemia (triglycerides ≥ 500 mg/dL and ≥ 880 mg/dL, respectively).^1–3^ FCS is a genetic form of sHTG that presents with higher fasting triglyceride levels (1,500 to 15,000 mg/dL compared with 500 to 2,000 mg/dL).^2^^,^^4–9^ It is an extremely rare form of sHTG,^2^ affecting approximately 0.0001% of the United States (US) population,^10^ compared with sHTG, which affects up to 2% of the population.^3^^,^^11^ Although sHTG and FCS present with similar symptoms, symptoms of FCS are more severe due to differences in underlying etiologies.^2^^,^^12^ Both sHTG and FCS are associated with an increased risk of significant adverse health outcomes, such as cardiovascular disease, diabetes, and pancreatitis, where hypertriglyceridemia-induced acute pancreatitis (AP) has been linked to higher illness severity, complication rates, and healthcare resource utilization.^13–18^

sHTG is associated with higher rates of all-cause pancreatitis and all-cause mortality compared to those without sHTG.^19^^,^^20^ The onset of acute pancreatic episodes can result in or amplify symptoms of sHTG, such as severe abdominal pain, gastrointestinal symptoms, fever, appetite loss, fatigue, and functional issues (ie, cognitive impairment).^21^^,^^22^ As a result, individuals experience a reduced health-related quality of life, with symptoms impacting their work life, social life, relationships, psychological well-being, and daily activities.^21^^,^^22^

The main treatment goal of sHTG is to lower elevated triglyceride levels. Dietary restrictions (ie, maintenance of a low-fat diet) are a mainstay nonpharmacologic treatment option; however, stabilization of healthy triglyceride levels is rarely achieved with diet alone.^14^^,^^23^ Lipid-lowering pharmacotherapies (eg, fibrates) may be administered alongside dietary modifications, yet they have a delayed onset of action and may not lower triglyceride levels effectively.^14^^,^^23^^,^^24^ Recently, olezarsen—a self-administered, auto-injected antisense oligonucleotide pharmacotherapy—was approved by the US Food and Drug Administration (FDA) and the European Medicines Agency for the treatment of FCS, for use as an adjunct to diet.^25–28^

In order to determine treatment effectiveness of novel therapies like olezarsen, the symptom items of the FCS-Symptom and Impact Scale (FCS-SIS)^29^^,^^30^—developed for assessment of the most severe form of sHTG, FCS—were included in clinical trials of sHTG as exploratory endpoints to assess sHTG symptoms (eg, CORE, NCT05079919;^31^ and CORE-2, NCT05552326^32^) due to the similarities in the clinical presentations of FCS and sHTG. The FCS-SIS is a 17-item patient-reported outcome (PRO) measure that assesses common FCS symptoms (4 items) and impacts (13 items) that have been identified as the most important and relevant concepts to patients with FCS.^29^^,^^30^ The 4 symptom items of the FCS-SIS include abdominal pain, physical fatigue, difficulty thinking, and diarrhea^29^^,^^30^—all of which are symptoms of sHTG.^33^

However, additional research is needed to further characterize the appropriateness of the 4 FCS-SIS symptom items in the context of sHTG. This qualitative interview study aimed to evaluate the content validity of these symptom items in adults with sHTG and to determine whether these items are fit-for-purpose for use in the entire sHTG population.

## Methods

### Interview participants

Individuals were recruited across several recruitment channels (eg, social media outlets, proprietary databases) by Opinion Health, a specialist recruiter of hard-to-reach patient groups. Eligible participants were required to be ≥ 18 years of age, have a clinician-confirmed diagnosis of sHTG ≥ 3 months prior to screening, and live in the US. Eligible participants also reported fasting triglycerides of ≥ 500 mg/dL (5.7 mmol/L) within the past 6 months and reported experiencing ≥ 2 sHTG symptoms (ie, abdominal pain, diarrhea, physical fatigue, difficulty thinking, AP, or other self-reported symptoms related to sHTG spontaneously described by participants) within the past month. Individuals were excluded if they met any of the following criteria at the time of screening: diagnosis of FCS, Crohn’s disease, ulcerative colitis, or irritable bowel syndrome; history of heart attack, stroke, or “mini stroke” (ie, transient ischemic attack) or receipt of cancer treatment within the past 6 months; diagnosis of diabetes mellitus (DM) in the past 3 months; and current participation in a clinical trial related to DM or triglyceride-lowering therapies. Considering the high prevalence of AP and DM among individuals with sHTG, recruitment subtargets were pursued for the recruitment sample, ensuring a balance of participants with and without a DM diagnosis and approximately one-third with a history of an AP event in the past year. Prior to interviewing, patient demographic and clinical data were obtained by Opinion Health, and patients provided confirmation of sHTG diagnosis and fasting triglyceride levels from their most recent lipid panel results.

Study materials were reviewed and approved by RTI International’s institutional review board (Federal-Wide Assurance #3331) before any potential participants were recruited for or consented to the study. Verbal informed consent was obtained from all study participants prior to the initiation of interviews, and the study was conducted in accordance with the Declaration of Helsinki.

### Interview procedures

Two rounds of remote hybrid qualitative interviews scheduled for up to 60-minutes each—comprising both concept elicitation and cognitive debriefing within a single interview—were conducted in English by experienced RTI Health Solutions (RTI-HS) interviewers using a study-specific, semistructured interview guide. Due to the consistency of participant feedback across the 2 rounds of interviews, results were reported in aggregate, unless otherwise specified.

Interviews began with concept elicitation followed by cognitive debriefing of the FCS-SIS symptom items. During concept elicitation, participants were asked general, open-ended questions to describe their initial experiences with sHTG and its symptoms and were asked to report their most bothersome symptom and the symptom they considered most important to improve. Participants were then asked targeted, open-ended questions to gather information pertinent to the research objectives, including the symptoms and impacts of sHTG. Participants initially reported their symptoms spontaneously, after which the 4 FCS-SIS symptoms were probed if not previously mentioned. During cognitive debriefing, participants were asked to provide feedback on the 4 symptom items of the FCS-SIS, including feedback on their relevance, interpretability, answerability, and comprehensiveness, as well as perceptions of meaningful change. The FCS-SIS symptom items (ie, measures of abdominal pain, difficulty thinking, physical fatigue, and diarrhea) are each rated using a 0-to-10 numeric rating scale (0, no abdominal pain/physical fatigue/difficulty thinking/diarrhea; 10, worst possible abdominal pain/physical fatigue/difficulty thinking/diarrhea); a 7-day recall period was assessed, aligning with that used in CORE/CORE-2.^31^^,^^32^ All interviews were audio recorded, and transcripts were generated to facilitate data analysis.

### Analyses

Using both the transcripts and field notes, dominant trends were identified in each interview and then compared with the results of all interviews to generate themes or patterns in the way participants described their experiences.^34^^,^^35^ Concept saturation, or the point at which no new information was elicited from participants,^36^ was evaluated with respect to reported symptoms. Analysis of the interview data was conducted according to a qualitative analysis plan and was facilitated by coding in Excel. Data were reported descriptively, and no statistical comparisons were conducted.

## Results

### Participant characteristics

A total of 20 participants were interviewed (10 per interview round). Most were female (n = 12 [60%]) and White (n = 11 [55%]), and the average age was 38.4 years (Table 1). Approximately half (n = 11 [55%]) had been diagnosed with DM. Most participants had a history of AP and had experienced an AP attack within the past 5 years (both n = 14 [70%]), and 40% (n = 8) had experienced an AP attack within the prior year. The mean triglyceride level of participants was 1,471.7 mg/dL. Symptoms most frequently identified by participants in the past month were physical fatigue (n = 18 [90%]), difficulty thinking (n = 14 [70%]), and diarrhea (n = 12 [60%]); 60% of participants (n = 12) reported their physical activity level as moderately active (eg, carrying light loads, participating in low-intensity exercise; some strenuous/physically challenging activity) or very active (eg, participating regularly in vigorous physical activity/exercise; frequent strenuous/physically challenging activity).

**Table 1.** Participant’s self-reported characteristics.

Characteristic	Total, N = 20
Current age, mean (SD) [range], years	38.4 (11.8) [26–59]
Age at sHTG diagnosis, mean (SD) [range], years	33.4 (7.6) [22–51]
Gender identity, n (%)	
Female	12 (60.0)
Male	8 (40.0)
Race and ethnicity, n (%)^ a^	
White	11 (55.0)
Hispanic, Latin American, Latino, or Latinx	4 (20.0)
African American or Black	3 (15.0)
Alaska Native, American Indian, Native American	1 (5.0)
Asian or Asian American	1 (5.0)
Middle Eastern or North African	1 (5.0)
Other: Jewish	1 (5.0)
Education level, n (%)	
Some high school	1 (5.0)
High school or equivalent	1 (5.0)
Some college	7 (35.0)
College degree	6 (30.0)
Graduate education	5 (25.0)
Employment status, n (%)	
Full time	11 (55.0)
Part time	2 (10.0)
Disability	2 (10.0)
Not employed/retired (on leave)	5 (25.0)
DM diagnosis, n (%)	11 (55.0)
Acute pancreatitis experience, n (%)	
Within the past year	8 (40.0)
Within the past 1-5 years	6 (30.0)
Never	6 (30.0)
History of AP	14 (70.0)
Triglyceride levels, mean (SD) [min, max], mg/dL	1,471.7 (1,268.1) [536, 5680]
Symptoms experienced in the past month, n (%)^ a^	
Physical fatigue	18 (90.0)
Difficulty thinking	14 (70.0)
Diarrhea	12 (60.0)
Abdominal pain	2 (10.0)
Heart palpitations	1 (5.0)
Currently on a low-fat diet	
Yes	14 (70.0)
No	6 (30.0)
Currently on a lipid-lowering diet (e.g., consume foods with omega-3 fatty acids, high fiber)	
Yes	15 (75.0)
No	5 (25.0)
Physical activity level	
Very active	3 (15.0)
Moderately active	9 (45.0)
A little active	5 (25.0)
Sedentary	3 (15.0)

Abbreviations. AP, acute pancreatitis; DM, diabetes mellitus; SD, standard deviation; sHTG, severe hypertriglyceridemia.

^a^ Multiple response item.

### Concept elicitation

#### Symptoms of sHTG

**Figure 1.**
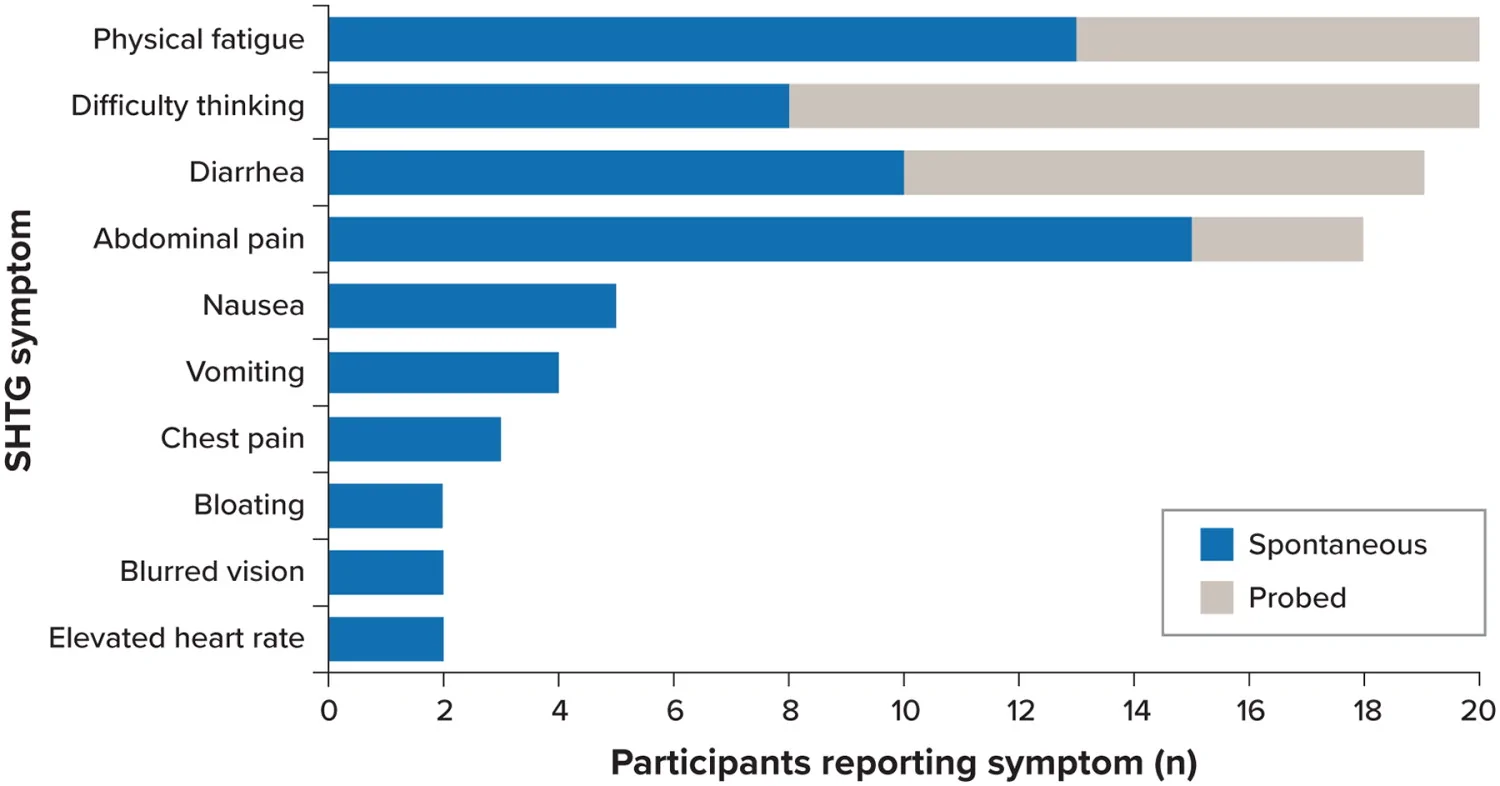
Symptoms of sHTG. Abbreviation. sHTG, severe hypertriglyceridemia.

The most frequently reported symptoms that sHTG participants were currently experiencing, based on both spontaneous reports and the total number of reported symptoms, included difficulty thinking, physical fatigue, diarrhea, and abdominal pain (Figure 1). These are the 4 symptoms comprising the FCS-SIS items, which were spontaneously mentioned in the first 2 interviews conducted. The first round of interviews captured most sHTG-related concepts; although new concepts were reported during the second round of interviews, no key symptoms representative of the overall sHTG experience were identified, indicating that concept saturation was achieved. Representative quotes of participants describing their reported sHTG symptoms are presented in Table 2.

**Table 2.** Representative quotes of reported sHTG symptoms.

Symptom	Representative quotes
Difficulty thinking	“*I’ve started taking vitamin D and vitamin B12, and it does seem to be helping*.” (IDI 3)
	“*I just have a common thought at times when I try to speak to somebody and I can’t think of the person’s name…this difficulty thinking is more like a brain fog all of a sudden, things like that, I haven’t experienced anything like that*.” (IDI 2)
	“*My brain is…I have the…it’s almost like having a wet brain. We’re just like, oh my God, this, and then it goes away after a while*.” (IDI 20)
Physical fatigue	“*It is just a chronic fatigue to a point where you just really don’t even want to move at times…It’s pretty much constant*.” (IDI 3)
	“*I will just be very sluggish [between the pancreatitis attacks]…closer to an attack…it’s more severe…I can’t really do anything. But other times, I can still function and get my things done*.” (IDI 5)
	“*The fatigue was more, because it was [during] a pancreatic attack…I was really, really tired, and weak to the bones*.” (IDI 16)
Diarrhea	“*Oh, absolutely…there’s no substance to it at all. It’s just oil and water. It just runs straight out*.” (IDI 14)
	“*If I eat a very fatty-type food, like in the afternoon it will be a photo finish to the potty and it is definitely triggered by food choices or stress…That’s almost an everyday thing*.” (IDI 18)
	“*When I [am about to] have a pancreatitis [attack]… I’ll see it in the stool…It’ll… have a terrible odor…it’s one of the other first signs where I’m like, uh-oh*.” (IDI 20)
Abdominal pain	“*[Abdominal pain is] a daily thing…it just feels like someone’s constantly pressing down…[during an attack] it’s more severe…it will just hurt to breathe, to move*.” (IDI 5)
	*“And my daily abdominal pain, I can handle it. I have abdominal pain right now because I drank some water. But when it’s severe, I feel like there’s a sword in me. There’s a dagger in between my shoulder blades*.” (IDI 6)
	“*It’s usually pretty sharp. It lasts a variable amount of time. I do have a variety of different meds that I take for it*.” (IDI 17)
Nausea	“*On a normal day, it’s more queasy…Like, I’ll just kind of feel like I’m off…But when it’s severe, I can’t look at food. I can’t look at water*.” (IDI 6)
	“*So around the nausea it occurs with the escalation of, like, the symptoms. So I experienced occasional nausea, a general feeling of unwellness that I couldn’t just shake it off. I experienced chest tightness*.” (IDI 16)
	“*I have nausea too, but it’s like so normal to me that I don’t really think about it [when it occurs with pancreatitis]…I am nauseous a lot but I take Ondansetron so it doesn’t bother me*.” (IDI 18)
Vomiting	“And *when it’s [abdominal pain] really, really bad, I throw up a lot, and sometimes even in the hospital, they have a hard time getting me to stop vomiting*.” (IDI 1)
	“*A lot a lot of nauseousness, vomiting. Just overall general discomfort*.” (IDI 3)
	“*I’ll tell you when I’m having these attacks, I’m usually vomiting*.” (IDI 17)

Abbreviations. IDI, in-depth interview; sHTG, severe hypertriglyceridemia.

All participants (spontaneous/probed, n = 20 [100%]; spontaneous, n = 8 [40%]) reported difficulty thinking as a symptom of their sHTG and noted problems associated with concentration, following conversations, and memory; several participants used the terms “brain fog” and “wet brain” to illustrate their experience. Participants commented on the episodic nature of this symptom; decrements and improvements in their thinking often occurred without precedent. However, participants occasionally observed that changes in their ability to think corresponded with changes in their diet or nutrition (eg, improvement in thinking when taking vitamin supplements). Although the frequency of this symptom varied from daily to monthly, those who experienced it during a pancreatitis event noted that its severity substantively increased compared with periods of difficulty thinking outside an event.

Similarly, all participants (spontaneous/probed, n = 20 [100%]; spontaneous, n = 13 [65%]) reported experiencing physical fatigue as a symptom of sHTG, commenting on feeling “sluggish” and “weak to the bones,” such that they were unable to move. When asked about the frequency of their fatigue, descriptions varied, ranging from a “chronic” to “constant” experience. One participant who did not have prior experience with AP described their fatigue as an infrequent, episodic occurrence that happened less than once a month. Those with AP noted that worsening of physical fatigue was often an indicator of impending or ongoing pancreatitis events, during which the severity of physical fatigue increased.

Nearly all participants (spontaneous/probed, n = 19 [95%]; spontaneous, n = 10 [50%]) reported experiencing diarrhea associated with their sHTG—characterized as the sudden, urgent passing of loose stools that could also be oily or malodorous (“It’s just oil and water”). Some participants noted that this symptom occurred as often as almost daily to only during the periods immediately prior to, during, and after a pancreatitis event. However, most participants reported their diarrhea tended to occur between pancreatitis events; these between-event episodes were often associated with food choices (eg, fatty food, red meat) that triggered their diarrhea. Further, at least 1 participant noted that stress exacerbated their diarrhea symptoms.

Nearly all participants (spontaneous/probed, n = 18 [90%]; spontaneous, n = 15 [75%]) reported abdominal pain associated with sHTG. Although participants with a history of AP reported experiencing abdominal pain between and during pancreatitis events (specifically, AP attacks), a few noted experiencing abdominal pain exclusively during these events. In characterizing the quality of their abdominal pain, participants’ descriptions varied, noting the pain between attacks could feel like “someone’s constantly pressing down” or “pretty sharp,” and in a few cases, it radiated to other parts of the body (eg, shoulder). Some participants noted their abdominal pain was always present, whereas others shared it came and went and was exacerbated by what they ate (e.g., dairy foods). All participants who experienced an AP attack noted that their abdominal pain intensity increased substantially during the event; the 2 participants who reported they had not experienced abdominal pain had never experienced an AP attack.

A quarter of participants (n = 5 [25%]) described experiencing nausea, which was characterized as a “queasy” feeling that could vary in severity and could escalate symptoms such as chest tightness. Many participants noted that they would take medication to relieve symptoms. Additionally, some participants (n = 4 [20%]) experienced vomiting, which frequently co-occurred with nausea and abdominal pain or a general worsening of symptoms leading up to a pancreatitis event. Less common sHTG-associated symptoms that participants reported included chest pain (n = 3 [15%]) and elevated heart rate, abdominal swelling/bloating, and blurry vision (each, n = 2 [10%]). Participants also mentioned skin problems, anxiety, high blood pressure, heartburn, shortness of breath, and cardiovascular problems in general (each, n = 1 [5%]).

##### Most bothersome and important to improve sHTG symptoms

**Figure 2.**
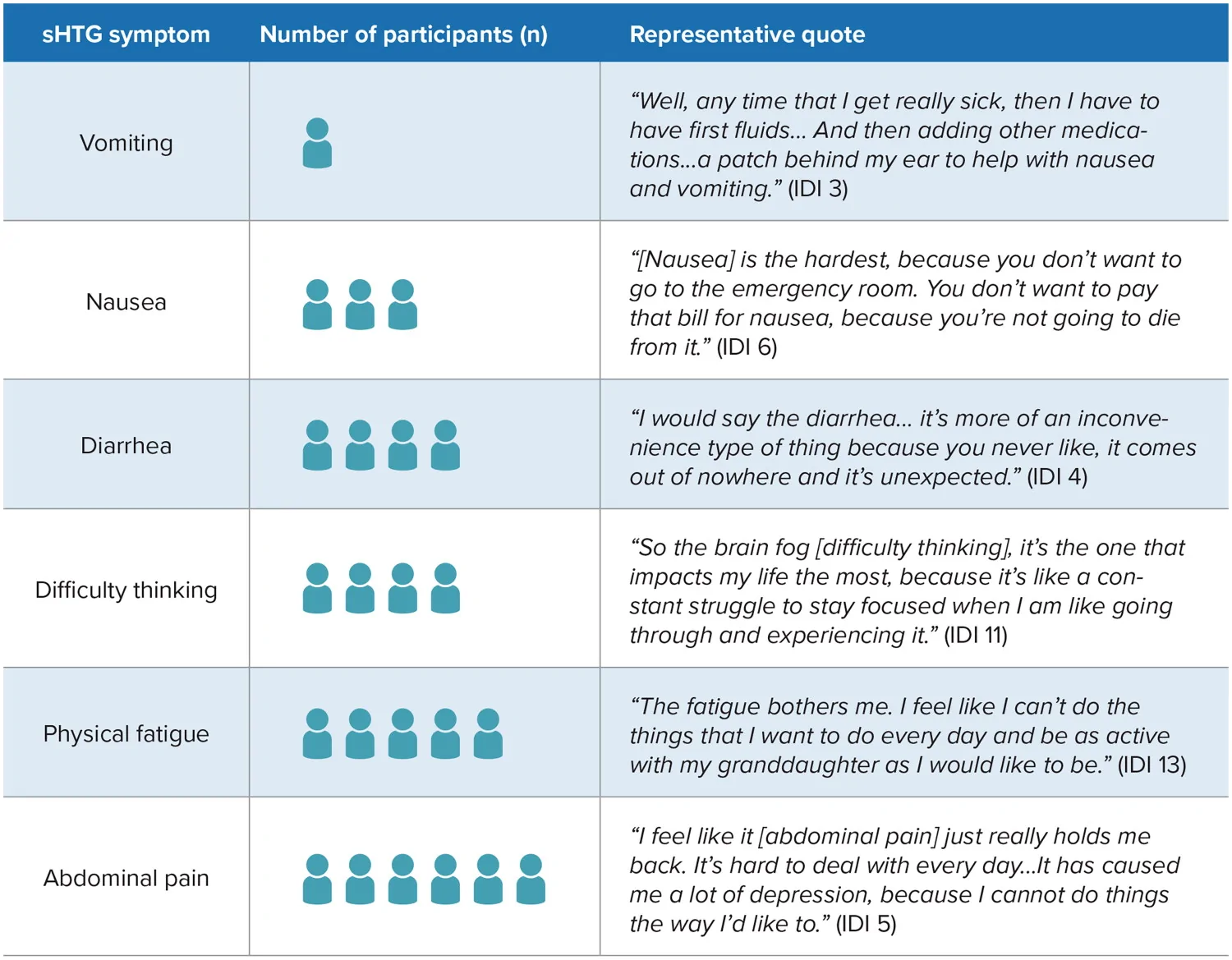
Most bothersome sHTG symptoms. Abbreviations. IDI, in-depth interview; sHTG, severe hypertriglyceridemia. Note: The total number of reported symptoms exceeds the number of participants (N = 20), as participants could report more than 1 symptom.

**Figure 3.**
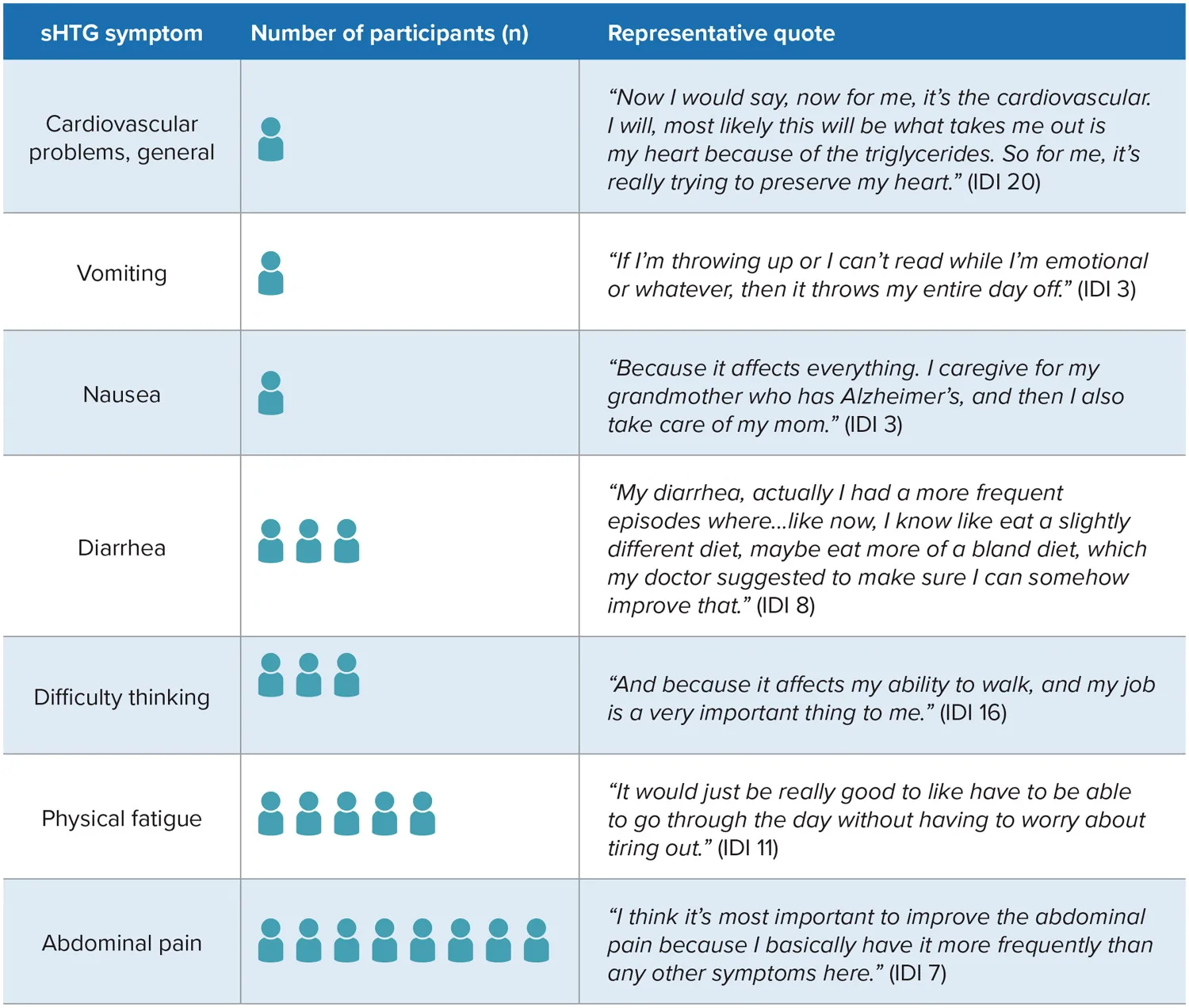
Most important to improve sHTG symptoms. Abbreviations. IDI, in-depth interview; sHTG, severe hypertriglyceridemia. Note: The total number of reported symptoms exceeds the number of participants (N = 20), as participants could report more than 1 symptom.

The most bothersome symptoms that were frequently reported by participants were abdominal pain (n = 6 [30%]), physical fatigue (n = 5 [25%]), difficulty thinking (n = 4 [20%]), and diarrhea (n = 4 [20%]) (Figure 2)—again, the 4 symptoms assessed by the FCS-SIS. When participants were asked about the sHTG symptoms they considered most important to improve, the same 4 symptoms were frequently reported: abdominal pain (n = 8 [40%]), physical fatigue (n = 5 [25%]), difficulty thinking (n = 3 [15%]), and diarrhea (n = 3 [15%]) (Figure 3). Participants’ explanations for seeking relief from their symptoms that are most important to improve closely aligned with their reports of their most bothersome symptom. Specifically, participants indicated a desire to reduce both the frequency and severity of the symptoms they considered most important to improve, as they were the most pervasive and disruptive to their day-to-day activities (eg, work productivity, participating in physical activity) and were described as physically or emotionally distressing. Moreover, participants reported the desire to enjoy food without restriction and mitigate any potential burden their symptoms might pose to family members.

#### Impacts of sHTG

Participants reported impacts of sHTG across 4 key domains: daily activities, work productivity, social activities, and mood and emotions (representative quotes are presented in Table 3). All participants (n = 20 [100%]) noted that sHTG affected their daily activities, specifically noting the pervasive nature of sHTG that required adjustments to their lifestyles, including changes to diet, physical activity, and carrying out chores or other tasks. In addition, all participants (n = 20 [100%]) reported that sHTG interfered with their productivity at work, where some participants had to quit their jobs or medically retire due to frequent pancreatitis or the effects of elevated triglycerides on their health. Almost all participants (n = 18 [90%]) reported limitations in their ability to participate in social activities due to their sHTG, which resulted in missing important events or opportunities to socialize. Also, most participants (n = 17 [85%]) reported experiencing a negative impact on their mood and emotions, including fear and anxiety of experiencing sHTG symptoms in public as well as concern about the impact of sHTG on their health, longevity, and families in general.

**Table 3.** Impacts of sHTG.

Impact category	Participants reporting impact, n (%)	Representative quotes
Daily activities	20 (100.0)	“*Limiting food that I would normally enjoy eating as opposed to foods that I know I should be eating, as opposed to let’s say, for example, I want potato chips, I’ll have a yogurt instead. It has a distinct impact on diet choices along those lines*.” (IDI 14)
		“*I used to be involved in team sports, soccer, to be precise. And these days, I can’t participate on the field in the way I would love to because I get tired easily*.” (IDI 7)
		“*My food preparation has totally changed…I kind of got rid of a lot of the greasy foods and things like that… I try and eat a lot less carbohydrates. I try and eat a lot more seeds, nuts, olive oil, fish, things like that…It’s hard*.” (IDI 17)
Work productivity	20 (100.0)	“*I’m supposed to be there [at work], but now I’m having to clock-in late because…I ate lunch, and now I’m really sick [diarrhea]; that sort of thing…It does affect your productivity*.” (IDI 3)
		“*I had to take off work…it was just taking me too much time to complete simple tasks than I normally do*.” (IDI 11)
		“*Normally, I love it [working with horses]. But when… I’m so tired that I just want to go lay down in the hay pile instead of take the hay out to the horses, that’s the worst*.” (IDI 18)
Social activities	18 (90.0)	“*Well, it definitely limits what I can eat, and occasionally it limits how well I feel and to what extent I can do certain things, whether it is eating out…I think that’s always hard when you have things that people want you to do and you have to quietly decline*.” (IDI 3)
		“*Back to the social aspect there, and it’s the same thing…we go to a bar, right? I get fish and chips or chicken tenders. Now I’m like… I get a salad with no dressing and chicken. It’s not fun*.” (IDI 17)
		“*The fatigue…They [kids] want to go and play, and I can’t keep up…I want to be able to play with my kids and keep up with my kids and them have a mom to play with*.” (IDI 9)
Mood and emotions	17 (85.0)	“*Yeah. I have a lot of fear: fear about how much pain I’m going to be in, and worry about my family, and worry about even just dying and them not having me anymore*.” (IDI 1)
		“*So sometimes you kind of feel as if you’re living on borrowed time because anything could happen*.” (IDI 7)
		“*I think I may be worrying about the impact of the food on like how it’s going to impact my health… I do think it’s causing me a little bit anxiety…overall [when thinking] about [food choices and] the symptoms*.” (IDI 11)

Abbreviations. IDI, in-depth interview; sHTG, severe hypertriglyceridemia.

### Cognitive debriefing

**Figure 4.**
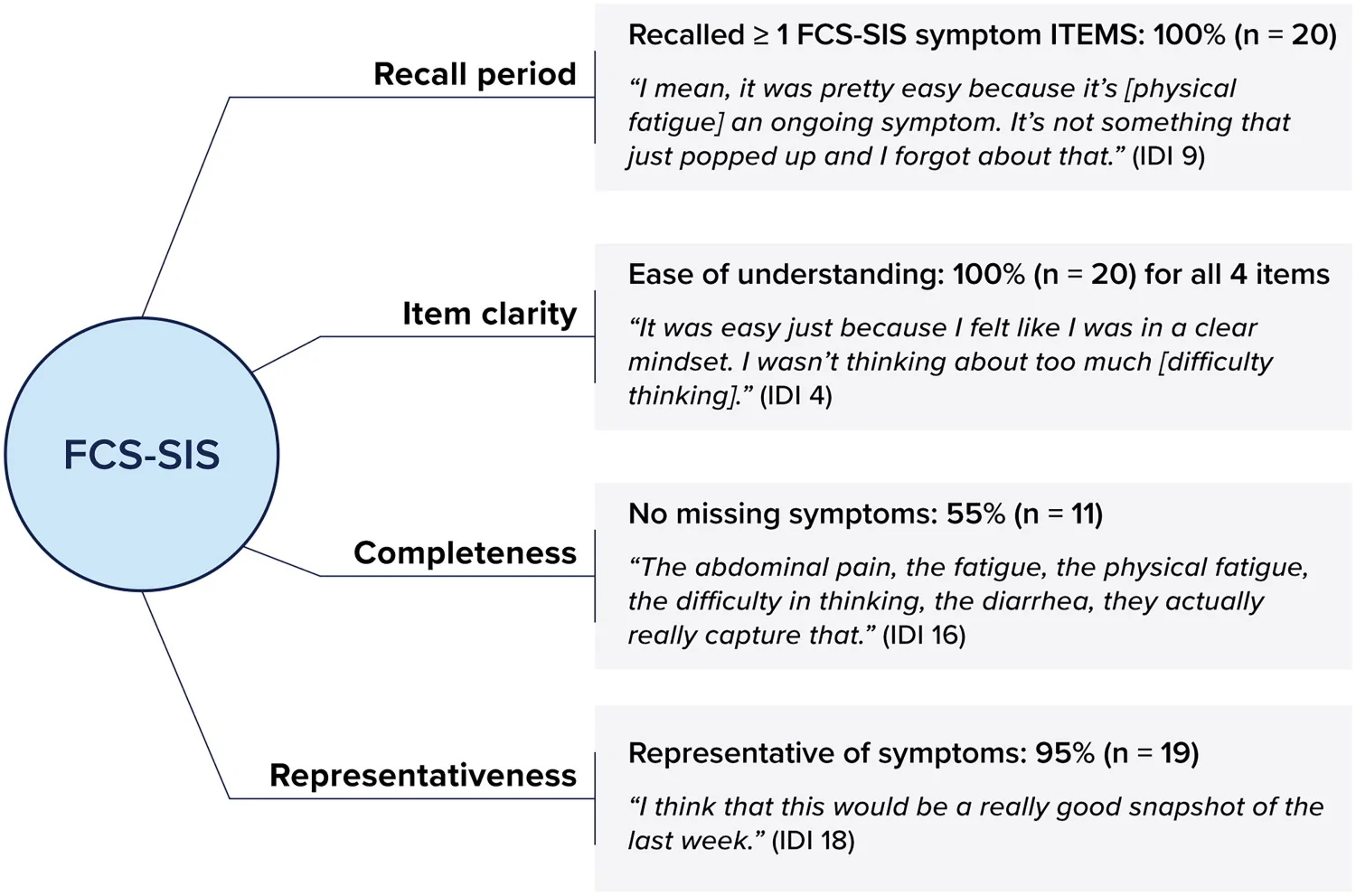
Cognitive debriefing summary and comprehensiveness of the FCS-SIS in sHTG. Abbreviations. FCS-SIS = Familial chylomicronemia syndrome-symptom and impact scale; IDI = in-depth interview; sHTG = severe hypertriglyceridemia.

To assess the clarity and ease of answering the FCS-SIS symptom items and to further support the relevance of the symptom items in sHTG, the items were debriefed with all participants. FCS-SIS symptom item feedback is summarized in Figure 4, and representative quotes describing symptom item feedback, including meaningful change improvements, are presented in Table 4.

**Table 4.** FCS-SIS symptom item feedback.

Symptom item	Easy to understand, n (%)	Representative quotes	1- to 2-point improvement would be meaningful, n (%)	≥ 3-point improvement would be meaningful, n (%)
Abdominal pain (Item 1)	20 (100.0)	“*My answer would be around 8…I’ve also experienced a very severe abdominal pain last week.*” (IDI 7)	13 (65.0)	5 (25.0)
		“*I thought about my worst pain…Then I thought about I haven’t [gone to a doctor or taken a stronger medicine] so I was***,** *like***,** *probably right around a 2***,** *[but if you take out the word worst] it would probably be an average.*” (IDI 17)		
Physical fatigue (Item 2)	20 (100.0)	“*I’d have to say a 5…I’ve been able to accomplish most of what I’ve needed to do…not really had to reschedule appointments, things to that nature. So overall, it’s been remotely productive.*” (IDI 3)	9 (45.0)	11 (55.0)
		“*10. I haven’t been able to get up and do anything.*” (IDI 6)		
Difficulty thinking (Item 3)	20 (100.0)	“*I would say a 6…I struggle the most with remembering and that I have to repeat a few things.*” (IDI 5)	15 (75.0)	5 (25.0)
		“*I would rate maybe at a 4…Once in a while, I lose track of what I’m talking about, memory issues, but that’s about it.*” (IDI 13)		
Diarrhea (Item 4)	20 (100.0)	“*So I would rate it 3…I remember this episode of diarrhea, which it lasted little over a day, I guess, but I was okay other days.*” (IDI 8)	12 (60.0)	8 (40.0)
		“*I’m going to say a 9…I had the worst, absolute worst diarrhea all the way till Thursday…The constant diarrhea, the amount of diarrhea, the smell of the diarrhea. All of it.*” (IDI 10)		

Abbreviations. FCS-SIS, Familial Chylomicronemia Syndrome-Symptom and Impact Scale; IDI, in-depth interview.

#### Recall period

All participants recalled at least 1 of the 4 symptoms of sHTG over the past 7 days, regardless of the recency of the last AP event, and found this period of time to be appropriate for measuring the concepts. Some participants commented on how some symptoms, such as physical fatigue and diarrhea, required more reflection to provide an answer due to these symptoms being “the norm” due to experiencing these symptoms frequently. For example, 1 participant noted that the sHTG symptoms they regularly experienced (ie, “the norm”) tended to become most pronounced within a 3-day window prior to experiencing a pancreatic event that would lead to hospitalization; as such, a briefer recall period was most relevant to this participant’s experience.

#### Item clarity

All participants (n = 20 [100%]) stated that the abdominal pain item was easy to understand and did not express difficulty responding to the item. Participants also stated that abdominal pain was relevant to sHTG, even if they themselves did not experience it. Of those who had experienced abdominal pain within the past 7 days (n = 16 [80%]) or more than 7 days ago (n = 2 [10%]), the majority (n = 13 [72%]) said that a 1- to 2-point improvement on the FCS-SIS would be meaningful; 5 participants indicated that at least a 3-point improvement on the scale would be necessary for it to be meaningful. In general, participants who rated their abdominal pain higher on the scale (ie, ≥ 5 points) required a greater point change to perceive the improvement as meaningful (mean change, 2.4 points), whereas those with lower pain ratings reported needing smaller improvements to experience meaningful change (mean change, 1.3 points).

All participants (n = 20 [100%]) found the physical fatigue item easy to understand and relevant to their experiences, with no difficulty in selecting a response. Two participants shared that it required more effort to reflect on their physical fatigue before selecting a response option, although they were ultimately able to provide an answer. All participants reported physical fatigue within the past 7 days (n = 20 [100%]); almost half (n = 9 [45%]) reported that a 1- to 2-point improvement would be meaningful, as any improvement was welcomed, whereas the others (n = 11 [55%]) noted that a minimum 3-point improvement was needed to confer a substantial improvement in their fatigue. In general, participants who reported higher fatigue levels on the scale (ie, ≥ 5 points) needed a greater improvement in their symptom ratings to perceive it as meaningful (mean change, 2.6 points), whereas those with lower fatigue ratings reported needing less point change (mean change, 1.3 points) to achieve meaningful improvement.

All participants (n = 20 [100%]) found the difficulty-thinking item easy to understand and provide an answer for and had experienced difficulty thinking within the past 7 days (n = 18 [90%]) or more than 7 days ago (n = 2 [10%]). Three-quarters (n = 15 [75%]) of participants reported that a 1- to 2-point improvement would be meaningful. The remaining participants (n = 5 [25%]) reported that at least a 3-point improvement was needed to experience a substantive improvement, as they would feel more confident in their thinking abilities. Furthermore, on average, participants who rated their difficulty thinking higher on the scale (ie, ≥ 5 points) needed a greater point improvement in their symptom ratings to consider it meaningful (mean change, 2.0 points), whereas those with lower fatigue ratings reported needing less change to experience a meaningful improvement in their symptoms (mean change, 1.1 points).

All participants (n = 20 [100%]) found the diarrhea item easy to understand as written and relevant to their experiences, including 2 participants who answered “0” but stated that they had experienced diarrhea, just not in the past 7 days; all remaining participants had experienced diarrhea within the past 7 days (n = 18 [90%]). Although 2 participants reported needing to pause to reflect on their answer, all participants were able to provide a response. Nearly two-thirds of participants (60%) reported that a 1- to 2-point improvement would be meaningful. The remaining participants (n = 8 [40%]) shared that at least a 3-point improvement would be meaningful to improving their symptom, as they would experience fewer interruptions in their day with needing to use the bathroom. Furthermore, on average, participants with higher ratings of diarrhea on the scale (ie, ≥ 5 points) required a greater point change to perceive a meaningful improvement (mean change, 2.7 points), whereas those with lower ratings reported needing less change to experience a meaningful improvement (mean change, 1.8 points).

#### Comprehensiveness and representativeness

At the end of the cognitive debriefing portion of each interview, participants were asked if any important symptoms relevant to sHTG were missing from the 4 symptom items. Of the 20 participants, more than half (n = 11 [55%]) reported that nothing was missing from the measure; however, 9 participants (45%) suggested adding other symptoms (Figure 4). Of the other symptoms that were suggested, nausea and chest pain (both, n = 3 [15%]) were most commonly reported and were symptoms experienced by participants. The remaining suggestions included skin issues, anxiety, blurry vision, heartburn, bloating, and high blood pressure (each, n = 1 [5%]).

When asked whether any of the symptoms (ie, abdominal pain, cognitive difficulties, physical fatigue, and diarrhea) were not relevant to their condition, 1 participant in round 2 who reported they had not experienced abdominal pain in the context of sHTG thought the item was not relevant because they had not personally experienced it. Two participants in round 2 also shared they were unsure whether difficulty thinking was relevant to their sHTG. Specifically, 1 participant was unsure about its salience, as their doctor had never asked them about it, whereas the other participant thought their thinking problems were a consequence of their abdominal pain experience. With the exception of the aforementioned instances, the remaining 17 participants all identified the 4 symptoms as relevant to sHTG. When asked to evaluate the representativeness and comprehensiveness of the 4 items as they relate to the assessment of sHTG, nearly all participants (n = 19 [95%]) described the items as being representative and providing a “good snapshot” of their symptom experience (Figure 4). One participant suggested that other factors, such as age and exercise, could also contribute to the symptoms.

## Discussion

This qualitative study revealed that the 4 symptoms evaluated in the FCS-SIS—abdominal pain, physical fatigue, difficulty thinking, and diarrhea—comprise the most frequently reported, bothersome, and important-to-improve symptoms in adults with sHTG. Participants described the onset or worsening of these 4 symptoms in the context of an AP event as well as the pervasive impact of these symptoms on their daily lives. Notably, participants described their AP events as exacerbations of symptoms beyond their typical severity that occurred simultaneously during an AP attack. Collectively, the results reflected the high prevalence and burden associated with the 4 symptoms comprising the FCS-SIS.

During debriefing of the FCS-SIS items, participants reported few, if any, challenges with respect to item language, recall, or response selection, and interpreted the items without difficulty. Additionally, patients were able to describe what would be required to attain a meaningful improvement in the FCS-SIS symptom items. Participants were globally in agreement that the symptom items were relevant to their experiences with sHTG, and each participant reported the presence (ie, score ≥ 0) of at least 1 of the 4 symptoms in the past week. Although some participants expressed a desire for the assessment of symptoms not included in the measure, no more than 3 individuals supported the inclusion of any single symptom (eg, nausea, chest pain). Further, all but 1 participant reported that they thought the 4 items were sufficiently reflective of their sHTG, which suggests the other symptoms that were identified were not essential to evaluate. Thus, no new sHTG symptoms were identified that were recommended to be added to the FCS-SIS, confirming the appropriateness of the 4 symptom items as exploratory endpoints in sHTG clinical trials.

In exploring potential differences in FCS-SIS symptom ratings, it was found that, on average, individuals who rated a symptom as more severe (≥ 5 points) required greater change in their total symptom ratings to attain meaningful improvement compared with participants who rated their symptoms < 5 points on the FCS-SIS items. As individuals with higher symptom scores may have more “room” to experience improvement relative to those who rated a symptom lower on the scale (eg, rated their symptom a 1 or 2), this observation may partially account for the noted trend. Further, in exploring any potential differences in FCS-SIS symptom ratings in individuals with and without a history of AP, it was found that those with a history of AP may require a higher total (all 4 symptoms) mean difference in symptom scores (ie, require greater change in symptom rating) to achieve meaningful improvement (total mean difference: 2.1 points) compared with those without AP (total mean difference: 1.5 points). However, in light of the small sample size, small differences, and qualitative nature of the study, it is challenging to interpret this difference or attribute this trend to differences in total symptom severity ratings associated with a history of AP (total symptom severity mean score among those with AP: 5.1 points; total symptom severity mean score among those without AP: 4.7 points).

This study was performed in accordance with the *Guideline for Good Clinical Practice*^37^ and the FDA patient-focused guidance on fit-for-purpose clinical outcome assessment methods,^38^ which highlights the importance of establishing the content validity of a measure to provide evidence that it is a fit-for-purpose assessment. Most of the symptoms reported by participants in this study are consistent with those captured by PRO measures that specifically assess symptoms of AP associated with sHTG, which include the Hypertriglyceridemia and Acute Pancreatitis Symptom Scale and the Hypertriglyceridemia and Acute Pancreatitis Dietary Behavior measure.^39^ Although overlapping symptoms have additionally been reported for sHTG and FCS previously,^2^ the severity of symptoms can differ based on variances in phenotypic features.^12^ Those with sHTG have some lipoprotein lipase activity to support triglyceride metabolism, whereas those with FCS are lipoprotein lipase deficient and therefore experience triglyceride levels much higher than individuals with sHTG.^40^^,^^41^

It is important to note that, due to the small sample size, which had an average age lower than that reported for the general sHTG population,^11^^,^^42^ and due to only selecting patients who reported at least 2 symptoms in the past month, study findings may not be generalizable to the broader sHTG population, including those patients who are asymptomatic. On the contrary, the qualitative study design was appropriate for the study objectives, and the sample was representative of the clinical trial population (eg, patients with and without history of AP and DM).

## Conclusion

sHTG is associated with a considerable symptom burden that impacts patients’ daily lives. This qualitative study provided content validation for the FCS-SIS symptom items in adults with sHTG. These items represented symptoms identified as most frequently reported, bothersome, and important-to-treat sHTG symptoms, thereby supporting their use in sHTG clinical trials and real-world evidence generation, providing patient insight into the symptoms and impacts of sHTG.

## Data Availability

Data are primarily in the form of transcripts and cannot be made available in order to protect participant privacy in accordance with the principles of the Belmont Report.

## Transparency

### Declaration of financial/other relationships

Asia Sikora Kessler and Aaron Yarlas are employees of Ionis Pharmaceuticals Inc. and hold stocks and/or options in Ionis. T. Michelle Brown, Bonita Basnyat, and Jennifer Dine are full-time employees of RTI Health Solutions, a subsidiary of RTI International, a nonprofit organization that conducts work for government, public, and private organizations, including pharmaceutical companies, which was retained by Ionis Pharmaceuticals Inc. to conduct the research that is the subject of this manuscript. Their compensation is unconnected to the studies on which they work.
